# New Amphiphilic Squalene Derivative Improves Metabolism of Adipocytes Differentiated From Diabetic Adipose-Derived Stem Cells and Prevents Excessive Lipogenesis

**DOI:** 10.3389/fcell.2020.577259

**Published:** 2020-11-04

**Authors:** Munkhzul Ganbold, Farhana Ferdousi, Takashi Arimura, Kenichi Tominaga, Hiroko Isoda

**Affiliations:** ^1^National Institute of Advanced Industrial Science and Technology (AIST)-University of Tsukuba Open Innovation Laboratory for Food and Medicinal Resource Engineering (FoodMed-OIL), Tsukuba, Ibaraki, Japan; ^2^Alliance for Research on the Mediterranean and North Africa (ARENA), University of Tsukuba, Tsukuba, Ibaraki, Japan; ^3^Faculty of Life and Environmental Sciences, University of Tsukuba, Tsukuba, Ibaraki, Japan

**Keywords:** squalene, derivative, ASC, diabetes, adipose-derived stem cell differentiation, adipocyte metabolism, adipocyte differentiation

## Abstract

Squalene (Sq) is a natural compound, found in various plant oils, algae, and larger quantity in deep-sea shark liver. It is also known as an intermediate of cholesterol synthesis in plants and animals including humans. Although evidences demonstrated its antioxidant, anticancer, hypolipidemic, and hepatoprotective and cardioprotective effects, its biological effects in cellular function might have been underestimated because of the water-insoluble property. To overcome this hydrophobicity, we synthesized new amphiphilic Sq derivative (HH-Sq). On the other hand, adipose-derived stem cells (ASCs) are a valuable source in regenerative medicine for its ease of accessibility and multilineage differentiation potential. Nevertheless, impaired cellular functions of ASCs derived from diabetic donor have still been debated controversially. In this study, we explored the effect of the HH-Sq in comparison to Sq on the adipocyte differentiation of ASCs obtained from subjects with type 2 diabetes. Gene expression profile by microarray analysis at 14 days of adipogenic differentiation revealed that HH-Sq induced more genes involved in intracellular signaling processes, whereas Sq activated more transmembrane receptor pathway-related genes. In addition, more important number of down-regulated and up-regulated genes by Sq and HH-Sq were not overlapped, suggesting the compounds might not only have difference in their chemical property but also potentially exert different biological effects. Both Sq and HH-Sq improved metabolism of adipocytes by enhancing genes associated with energy homeostasis and insulin sensitivity, *SIRT1*, *PRKAA2*, and *IRS1*. Interestingly, Sq increased significantly early adipogenic markers and lipogenic gene expression such as *PPARG*, *SREBF1*, and *CEBPA*, but not HH-Sq. As a consequence, smaller and fewer lipid droplet formation was observed in HH-Sq-treated adipocytes. Based on our findings, we report that both Sq and HH-Sq improved adipocyte metabolism, but only HH-Sq prevented excessive lipogenesis without abrogating adipocyte differentiation. The beneficial effect of HH-Sq provides an importance of synthesized derivatives from a natural compound with therapeutic potentials in the application of cell therapies.

## Introduction

Human adipose-derived stem cells (ASCs) are adult stem cells obtainable by liposuction procedure. By definition, ASCs present high capacity to proliferate after isolation without losing their stemness, at the same time potential to differentiate into mesodermal cell lineages, including adipocytes, chondrocytes, osteocytes, and myocytes under appropriate inducing condition.

The therapeutic and regenerative effect of ASCs on insulin resistance, obesity, and type 2 diabetes (T2D)-associated complications has been investigated for a variety of purposes (Mazini et al., 2020). For example, multiple infusion of ASCs in diabetes-induced rats reversed diabetes-related complications and protected tissue damages by alleviating inflammation (Yu et al., 2019). A recent study revealed that subcutaneous transplantation of ASC sheet prepared from non-diabetic mice into diabetic mice improved glucose intolerance (Ishida et al., 2020). Subcutaneous injection and local administration of human adipose-derived stromal vascular fraction cells showed a beneficial effect on microcirculation in ischemic diabetic feet and wound healing (Nie et al., 2011; Moon et al., 2019). Moreover, its application in tissue engineering and fat grafting in reconstructive surgery is being extensively explored (Naderi et al., 2014, 2017).

However, previous studies raised questions about the effectiveness of ASCs isolated from a diabetic and obese donor on the clinical use of stem cells because of the predisposition of impaired biological functions and altered transcriptomes due to its microenvironment, thus diminishing its therapeutic value and further cellular function in the recipient. Proliferation, viability, and migration capacity of ASCs from the obese environment are reduced, and its transcriptome is altered and inflamed (Vyas et al., 2019).

Squalene (Sq), a polyunsaturated hydrocarbon, is found ubiquitously in numerous plant oils (Owen et al., 2000; He et al., 2002; Shimizu et al., 2019) and algae (Kaya et al., 2011) and also larger quantity in deep-sea shark liver (Hernández-Pérez et al., 1997). Sq is also synthesized in plants and animals including humans as an intermediate of sterol synthesis such as cholesterol. Knockout murine in which upstream enzyme of Sq synthesis was disrupted presented lipid dystrophy, hepatic steatosis, and severe diabetic abnormalities, showing its indispensable physiological function in the organism (Yeh et al., 2018). Numerous evidences demonstrated its antioxidant, anticancer, hypolipidemic, and hepatoprotective and cardioprotective effects. Dietary Sq supplement (1%) prevented colon carcinogenesis in rats (Rao et al., 1998); rabbits fed with Sq (3%) for 7 weeks unexpectedly showed no more atheroma than the controls (Kritchevsky et al., 1953); moreover, in Sq feeding (900 mg/d) in human studies, serum triglyceride and cholesterol contents were unchanged (Strandberg et al., 1990). Interestingly, contrary to the expected, in the same studies, Sq feeding did not increase serum cholesterol level, whereas serum Sq level was augmented 17 times in humans after 30 days. These findings altogether suggest that Sq might be accumulated in the serum because of the lack of efficient absorption, even though its biological effect is beneficial.

Naturally, Sq is characterized as a hydrophobic lipid, and its biological properties in cellular function might have been underestimated because of the low solubility. Thus, to overcome this water-insoluble property, a recent study proposed a lysosome-embedded Sq structure and showed its preferential cytotoxic effect on LAN5 cancer cells in their preliminary study (Costa et al., 2018).

We have recently synthesized Sq derivative (HH-Sq) (Sasaki et al., 2020), which gained amphiphilic property compared to Sq. In this *in vitro* study, we explored the effect of HH-Sq compared to Sq on adipocyte differentiation of ASCs derived from diabetic donors to determine whether the gained hydrophilicity of the derivative can promote Sq’s biological effects and eventually be used in therapeutic application.

## Materials and Methods

### Chemicals

**FIGURE 1:**
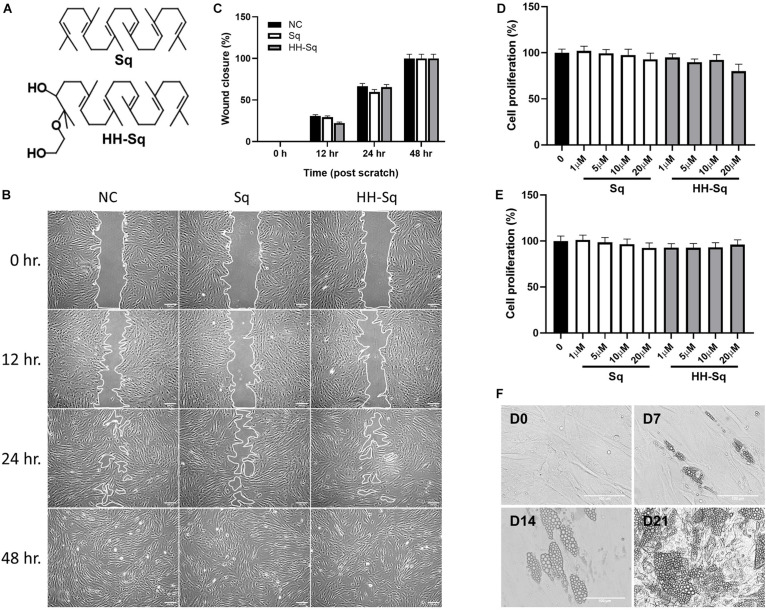
Effect of Sq and HH-Sq on dASCs migration, proliferation, and differentiation capacity of dASCs. **(A)** Chemical structure of squalene (Sq) and its derivative 2-(2-hydroxyethoxy)-3-hydroxysqualene (HH-Sq). **(B)** Representative images of scratch wound healing at indicated timepoints after treatment with 1 μM of Sq and HH-Sq. Cell-free area was outlined. Scale bar indicates 200 μm. **(C)** Cell-free area was measured from the microscopic images using ImageJ and represented as wound closure in percentage. NC indicates non-treated negative control. Cells were treated with different concentration of Sq and HH-Sq for **(D)** 48 h and **(E)** 72 h. Proliferation rate was compared to non-treated group. Data are shown as mean ± SEM (*n* = 5–6/dose). Statistical difference was calculated using one-way ANOVA followed by Dunnett test. **(F)** Representative light microscopic images at indicated timepoints (days) after the induction of adipocyte differentiation in dASCs. Scale bar indicates 100 μm.

Squalene was purchased from Fujifilm Wako (Tokyo, Japan). Sq mono ethylene glycol derivative, (2-(2-hydroxyethoxy)-3-hydroxysqualene) (HH-Sq), was synthesized as reported previously (Sasaki et al., 2020) and identified by spectral data (Figure 1A).

### Adipose-Derived Stem Cells Culture

Diabetic ASCs (dASCs) (T2D; 74-year-old Caucasian woman, body mass index = 25 kg/m^2^) was purchased from Lonza (Walkersville, MD, United States; Material number: PT-5008, tissue acquisition number: 29752). These cells were isolated from donated human tissue after obtaining permission for their use in research only by informed consent or legal authorization. Cells were cultured in ASC growth medium (ADSC-GM BulletKit^TM^) obtained from Lonza containing ASC basal medium supplemented with 10% fetal bovine serum, 1% L-glutamine, and gentamicin-amphotericin B (GA-1000) according to the manufacturer’s instruction at 37°C in 5% CO_2_ humidified incubator. Cells are subcultured 5,000 cells per cm^2^ as recommended by the provider.

### Adipogenic Differentiation of dASCs

Cells were seeded at a density of 3 × 10^4^ cells per cm^2^ in the preadipocyte growth medium (PGM-2^TM^ BulletKit; Lonza, Walkersville, MD, United States). Once cells reach confluence greater than 80%, adipogenesis is induced by changing medium to adipogenic differentiation medium (ADM). According to the manufacturer’s instruction, ADM was prepared by adding rhInsulin, dexamethasone, IBMX, and indomethacin into PGM. The medium was changed every 10 to 12 days.

### Cell Proliferation Assay

Cell proliferation was determined using the CellTiter 96^®^ AQueous One Solution Cell Proliferation Assay (Promega, Madison, WI, United States). Briefly, cells were seeded at a density of 1 × 10^4^ cells per well in 96-well plate for overnight and then treated with increasing concentration of Sq and HH-Sq. After 24 and 48 h of incubation, 20 μL per well of CellTiter 96^®^ AQueous One Solution Reagent was added and incubated for 3 h. Soluble formazan produced was measured by absorbance at 490 nm using a microplate reader (Varioskan LUX).

### Cell Migration/Scratch Assay

Cells were grown in ADSC-GM until confluence in 6-well plate and then washed with phosphate-buffered saline (PBS) two times, scratched using a sterile 10 μL micropipette tip, and incubated in ADSC-GM with treatment; the microscopic photo was taken at several timepoints; the cell-free area was measured using ImageJ.

### Lipid Droplets Staining

Cells were seeded on a chamber slide, and adipogenesis differentiation was induced in the presence or absence of Sq and HH-Sq as described above. Lipid droplets (LDs) formed after differentiation were stained by fluorescent neutral lipid dye 4,4-difluoro-1,3,5,7,8-pentamethyl-4-bora-3a,4a-diaza-s-indacene (BODIPY 493/503; Invitrogen, Eugene, OR, United States), mounted with ProLong^TM^ Diamond Antifade Mountant with DAPI (Invitrogen, Eugene, OR, United States) for staining nucleus, and fluorescence images were obtained by using Leica TCS SP8 confocal microscope. For quantification of LD accumulation, cells were seeded in a 96-well plate and induced adipogenesis differentiation. After washing with PBS, 5 μL of AdipoRed^TM^ Assay Reagent (Lonza) was added in each well containing 200 μL PBS and incubated for 10 min at room temperature. Fluorescence with excitation at 485 nm and emission at 572 nm was measured by using a microplate reader (Varioskan LUX).

### Microarray Analysis

Total RNA was extracted as above, and 250 ng of RNA was subjected to strand synthesis using GeneChip 3′ IVT PLUS Reagent Kit (Affymetrix Inc., Santa Clara, CA, United States) and Affymetrix^®^ 3′ IVT Array Strips for GeneAtlas^®^ System (GeneChip^®^ HG-U219) following the manufacturer’s protocol. Probe cell intensity values were obtained by GeneAtlas^TM^ Imaging Station; CEL files from probe sets were generated by the Expression Console (Affymetrix) and then analyzed by Transcriptome Analysis Console v4.0 (Affymetrix, Japan) as described previously (Ganbold et al., 2019). We have used three technical replicates of each sample (IN, Sq, and HH-Sq) that represent the same hybridization mixture applied to three independent arrays. Gene expression values refer to Tukey bi-weight average of gene level intensity of all the replicates in a condition. A threshold value of fold change ≥±1.2 (in linear space) and *p* < 0.05 [one-way between-subject analysis of variance (ANOVA)] employed by the manufacturer’s analysis package was set to identify differentially expressed genes (DEGs). Downstream analyses were conducted using the functional annotation clustering of DAVID Informatics Resources 6.8^1^ (Huang et al., 2009), and GSEA’s Molecular Signatures Database v7.1^2^ (Subramanian et al., 2005) for gene ontology (GO) and biological process (BP) clustering. GOs with *p* < 0.05 in DAVID (modified Fisher exact test with default EASE score threshold 0.1) were considered as significantly enriched. The microarray dataset was deposited in the NCBI Gene Expression Omnibus (GEO) and is accessible with the accession number GSE153391^3^.

### ATP Assay

ATP production was determined by the “cell” ATP assay luminescent reagent version 2 (CA2-50, Toyo Benet, Japan). Cells were seeded in white clear-bottom 96-well plate, and induced adipogenesis. After 14 days, according to the manufacturer’s protocol, the luminescent reagent (100 μL/well) was added and followed by 1 min on shaker and 10 min for incubation in dark at room temperature. Luminescence amount (RLU) was measured by microplate reader (Varioskan LUX) and normalized to cell number using Cell Count Normalization kit (Dojindo, Japan).

### Mitochondrial Staining by Rhodamine 123

Mitochondrial biogenesis was evaluated by rhodamine 123 assay. Cells were seeded in black clear-bottom 96-well plate and induced adipogenesis. At day 14 (D14), the supernatant was replaced by 50 μM rhodamine 123 (Wako, Japan) dissolved in Hanks balanced salt solution (Gibco) containing 20 mM HEPES (100 μL/well) and incubated at 37°C for 30 min. The supernatant was discarded and rinsed with the buffer two times before measuring rhodamine 123 fluorescence intensity [relative fluorescence unit (RFU)] using the excitation/emission at 485/525 nm by microplate reader (Varioskan LUX). RFU was normalized to cell number using Cell Count Normalization kit (Dojindo, Japan). After staining with rhodamine 123, cell nuclei were counterstained with 0.5 μg/mL Hoechst 33342 (Molecular Probes, United States) dissolved in PBS (100 μL/well) for 10 min at room temperature for fluorescent imaging using automated inverted microscope (IX83, Olympus).

### Glucose Uptake Assay

Glucose uptake was determined by Glucose CII test kit (Wako, Japan). Cells were seeded in 24-well plate and induced adipogenesis for 14 days. Differentiated adipocytes were starved in serum free medium for overnight. Next day, the medium was replaced with Krebs-Ringer-phosphate-HEPES (KRPH) buffer for glucose starvation. After 2 h of incubation, the buffer was replaced by KRPH containing 11 mM glucose with or without 1 nM insulin for 24 h. Glucose level in the supernatant was quantified by colorimetric assay according to the manufacturer’s instruction.

### Real-Time Reverse Transcription-Polymerase Chain Reaction Analysis

Cells were seeded and induced adipogenesis in a 6-well plate. Total RNA was extracted by using ISOGEN reagent (Nippon Gene, Toyama, Japan) following the manufacturer’s instruction. Complementary DNA was synthesized by reverse transcription from 1 μg of RNA using SuperScript^TM^ IV VILO^TM^ Master Mix (Invitrogen, Carlsbad, CA, United States). The resultant cDNA (100 ng) was then amplified by the predesigned TaqMan^®^ Gene Expression Assay and TaqMan^®^ Gene Expression Master Mix (Applied Biosystems, Carlsbad, CA, United States) on the 7500 Fast Real-Time PCR System. All predesigned primers were purchased from the Applied Biosystems. Gene expression was normalized to *GAPDH* as an endogenous housekeeping gene, and the relative expression level was calculated using the 2^–Δ^ ^Δ^ ^*Ct*^ method (*n* = 3).

### Capillary Immunoassay

Protein level was evaluated using ProteinSimple capillary immunoassay (Wes^TM^, ProteinSimple). dASCs induced adipogenesis in 6-well plate. Total protein was extracted at D14 using radioimmunoprecipitation assay buffer (Sigma-Aldrich, St. Louis, MO, United States) containing protease inhibitor cocktail (P8340, Sigma-Aldrich), and the protein concentration was quantified using 2-D Quant kit (GE Healthcare, Chicago, IL, United States). An equal amount (0.75 μg/μL) of protein diluted in sampling buffer (ProteinSimple) were loaded in 12- to 230-kDa, 25-lane plate (ProteinSimple) and run in WES WS3390 instrument following the manufacturer’s instruction. Primary antibodies were purchased from Abcam. Anti-rabbit and anti-mouse secondary antibodies of Detection module for chemiluminescence (ProteinSimple) were used. Imaging and peak area were obtained from Compass for SW software (ProteinSimple). Peak areas were normalized to GAPDH as loading control, and relative ratio was calculated compared to IN.

### Statistics

All data are shown as the mean ± SEM unless otherwise mentioned. Statistical analyses were conducted using GraphPad Prism 8.0. All data were assessed for normality (Shapiro–Wilk test) and kurtosis and skewness (D’Agostino-Pearson omnibus test). A one-way ANOVA followed by Dunnett *post hoc* test was performed to compare the treatment groups to the control group, whereas Tukey *post hoc* test was performed to compare among all three groups. Paired *t* test was used for within-group comparison at two different timepoints. A *p* < 0.05 was considered significant.

## Results

### Sq and HH-Sq Did Not Alter the Proliferative and Migratory Capacity of dASCs

Adipose-derived stem cells derived from T2D donor may have reduced or lost proliferation and migration potential leading to incapacity to differentiate (Pérez et al., 2015; Barbagallo et al., 2017; Vyas et al., 2019). Thus, we checked first if dASCs were maintaining its functions to proliferate and migrate as a sign of multipotent cells. After full confluence, cells in growth medium were subjected to a scratch/wound and then continued with the treatment of 1 μM Sq and HH-Sq for up to 48 h (Figure 1B). Non-treated (NC) cells were observed to migrate toward the cell-free area 12 h after the initiation of the wound and covered greater than 50% after 24 h and completely closed by 48 h. The wound closure rate in each treatment group reached 100% versus NC at the 48-hour time point (Figure 1C). Treatment with Sq and HH-Sq did not interfere with the migratory capacity of dASCs.

Moreover, expansion potential in culture is an important criterion for further use of stem cell source to differentiate into numerous cell lineages. Therefore, the cell proliferation rate of dASCs was evaluated in the presence of different dose of Sq and HH-Sq (Figures 1D,E), and both compounds did not show any inhibitory effect on the dASC proliferation rate until 20 μM of dose up to 72 h (48 h, *p* = 0.179; 72 h, *p* = 0.895). These results suggest that dASCs still preserve their proliferative and migratory ability, on which the treatment with Sq and HH-Sq did not show any inhibitory effect.

### Adipocyte Differentiated From dASCs After Induction

The primary role of stem cells from adipose tissue is to sustain adipocyte turnover by pooling newly differentiated adipocytes. However, evidences showing the capacity of dASCs to differentiate into adipocyte are still controversial (Pérez et al., 2015; Barbagallo et al., 2017). Thus, adipogenesis was induced in dASCs, and the appearance of LDs was observed 7, 14, and 21 days post-induction to explore the differentiation capacity and timing *in vitro* (Figure 1F). Only some of the cells began to show small LD formation at D7. Subsequently, by D14, the number of cells containing LDs occurred increasing along with the enlarged LD size leading the majority of cells engorged with LDs at D21. For further evaluation of the expression of genes, we chose dASCs differentiated on D14 as our model.

### Adipocyte-Induced dASCs Gene Expression Pattern Treated With Sq and HH-Sq

**FIGURE 2:**
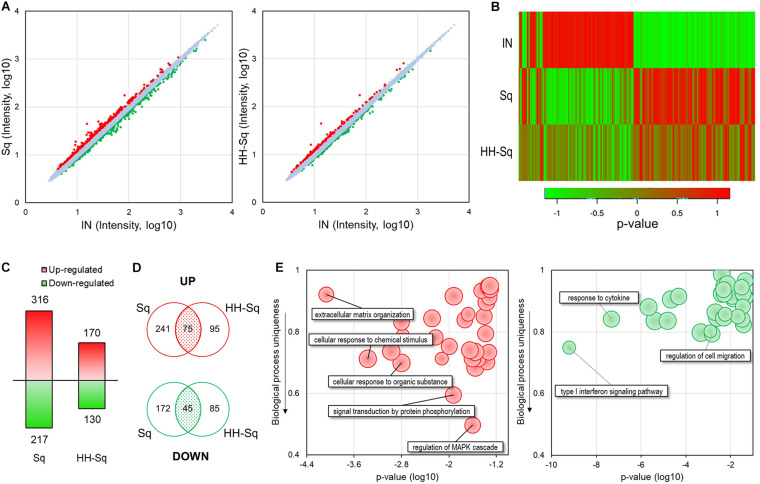
Microarray analysis of gene expression pattern in dASCs treated with Sq and HH-Sq at D14 after adipogenic induction. **(A)** Scatterplot of total gene expression intensity values in Sq- and HH-Sq-treated groups compared to the induced group (IN). Differentially expressed genes (DEGs) were marked in red for fold change ≥1.2 and in green ≤–1.2. **(B)** Heatmap of DEGs in each group was created by the *p* value using online tool (http://shinyheatmap.com/). **(C)** Number of DEGs in Sq- and HH-Sq-treated groups. **(D)** Venn diagram of up- and down-regulated genes in Sq- and HH-Sq-treated groups. **(E)** Biological process (top 30) of commonly up-regulated (left, red scatterplot) and commonly down-regulated (right, green scatterplot) genes were represented in scatterplots using REVIGO tool with modification (http://revigo.irb.hr/).

Next, we performed microarray analysis using dASCs of D14 after adipocyte differentiation treated with 1 μM Sq and HH-Sq and explored the gene expression profile. Intensity value of total gene probe set after treatment with 1 μM of Sq and HH-Sq compared to the non-treated adipocyte-induced control (IN) is shown (Figure 2A). Probes with a threshold of fold change ≥1.2 and *p* ≤ 0.05 were considered as DEGs and represented in the heatmap (Figure 2B). In the Sq-treated group, 532 genes were differentially expressed, of which 316 DEGs were up-regulated and 217 DEGs down-regulated, whereas in the HH-Sq-treated group, of 300 DEGs, 170 were up-regulated and 130 were down-regulated (Figure 2C). Subsequently, we analyzed if the respective up- and down-regulated genes in Sq and HH-Sq-treated groups were common or not using Venn diagram (Figure 2D). Interestingly, less than half of the up-regulated genes of HH-Sq (44%) and about one-fourth of Sq (23%) were overlapped, suggesting greater than the majority of genes were up-regulated in a compound-specific way. Similarly, genes overlapping in down-regulation in each group were lower than half of the total number.

Next, we identified main BPs involved in the commonly overexpressed (left, red) and underexpressed (right, green) genes applying GO analysis of DAVID and scattered the most relevant 30 processes according to their *p* value and uniqueness ratio differing from other BPs (Figure 2E). The most specific BP associated with GO terms mitogen-activated protein kinase (MAPK) cascade regulation (GO:0043408), signal transduction by protein phosphorylation (GO:0023014), and cellular response to chemical stimulus (GO:0070887) were up-regulated by both compounds. Applying the criteria of *p* > 0.05 and containing more than two genes, none of the GO terms among the top 30 BPs involving the common down-regulated genes was specific to adipogenesis (uniqueness ratio >0.5), signifying both Sq and HH-Sq do not have any particular common inhibitory effect on the adipogenesis process of dASCs.

### BPs Affected by Sq and HH-Sq Treatment in Adipogenesis of dASCs

**FIGURE 3:**
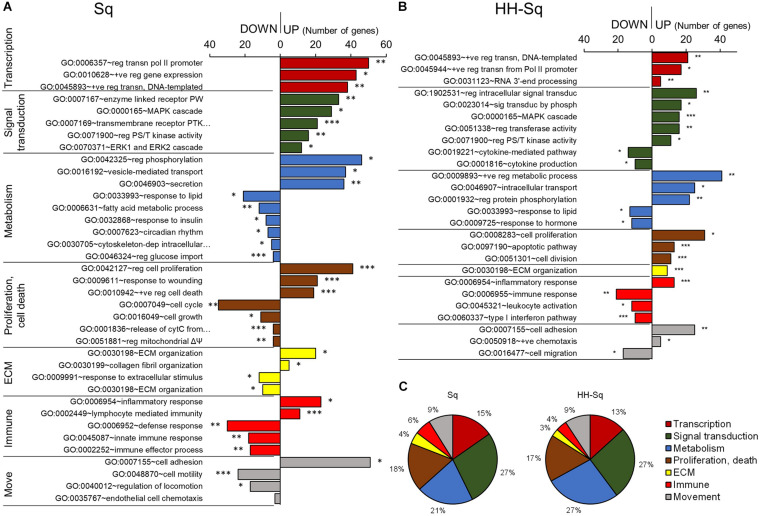
Gene ontology and biological process analysis in Sq and HH-Sq. Biological processes (BP) qualifying *p* < 0.05 (modified Fisher exact test) and more than 2 genes were extracted from GOTERM_BP_FAT analyzed by DAVID for **(A)** Sq and **(B)** HH-Sq treatments, and BP classification by the number of genes involved in down-regulated and up-regulated process is shown. *p < 0.05, **p < 0.01, and ***p < 0.001 vs. IN. Abbreviation: move, movement; reg, regulation of; +ve, positive; transn, transcription; PW, signaling pathway; PTK, protein tyrosine kinase; PS/T, protein serine/threonine; cytC, cytochrome C; ΔΨ, membrane potential; ECM, extracellular matrix. **(C)** Pie graph showing the ratio of number of up-regulated genes included in each cellular process category.

Next, to investigate the BP specifically affected by the compound, we identified all BP-qualifying *p* value greater than 0.05 and containing more than two genes specifically enriched in GO BP FAT terms in Sq and HH-Sq-treated groups and then classified into transcription, signal transduction, metabolism, proliferation and cell death, and extracellular matrix-, immune- and movement-related processes according to their relevance (Figures 3A,B). Conversely, large general terms such as regulation of metabolic process (GO:0009893) and regulation of signaling (GO:0023051) were excluded, and terms that are repetitive or similar were merged. Globally, transcriptional activity increased in both treatment groups, as shown by the increased number of genes associated with transcription of polymerase II promoter (GO:0006357) and positive regulation of DNA-templated transcription (GO:0045893); however, the number of genes enriched was two times higher in Sq group.

Among signal transducing events, MAPK pathway, extracellular signal-regulated protein kinase 1/2 cascade, and serine-threonine kinase activity-related gene expression were increased in Sq followed by cell proliferation regulatory and response to wounding processes activation. These results suggest that the overall MAPK cascade was activated to regulate cell cycle and proliferation during adipogenic differentiation. Hence, metabolic responses, including fatty acid metabolic process (GO:0006631), response to insulin (GO:0032868), and regulation of glucose import (GO:0046324), and cell cycle and growth process were down-regulated.

HH-Sq-treated cells have been found to not only up-regulate similar pathways such as MAPK cascade and serine-threonine kinase activity but also to directly over activates intracellular signal transduction (GO:1902531) and signal transduction by phosphorylation (GO:0023014) compared to Sq-treated cells. In addition, the HH-Sq treatment enhanced positive regulation of metabolic process (GO:0009893) and intracellular transport (GO:0046907). Interestingly, cytokine-mediated pathway (GO:0019221) and cytokine production (GO:0001816) were down-regulated, providing another HH-Sq-specific regulation. Inflammatory cytokines secreted by adipocytes in obese and diabetic environment are thought to be increased and promote systemic level of circulating proinflammatory cytokines, thereby contributing to insulin resistance development (Makki et al., 2013; Odegaard and Chawla, 2013). Collectively, the resulting changes show HH-Sq’s more favorable effect in alleviating disease-related molecular impairment.

All up-regulated DEGs classified into the cellular processes in Sq and HH-Sq by the number of genes showed a similar ratio suggesting the derivative HH-Sq exerts biological effect, which is comparable to its mother molecule (Figure 3C).

### Sq and HH-Sq Promoted Preadipocyte Differentiation Transcription Regulators

**FIGURE 4:**
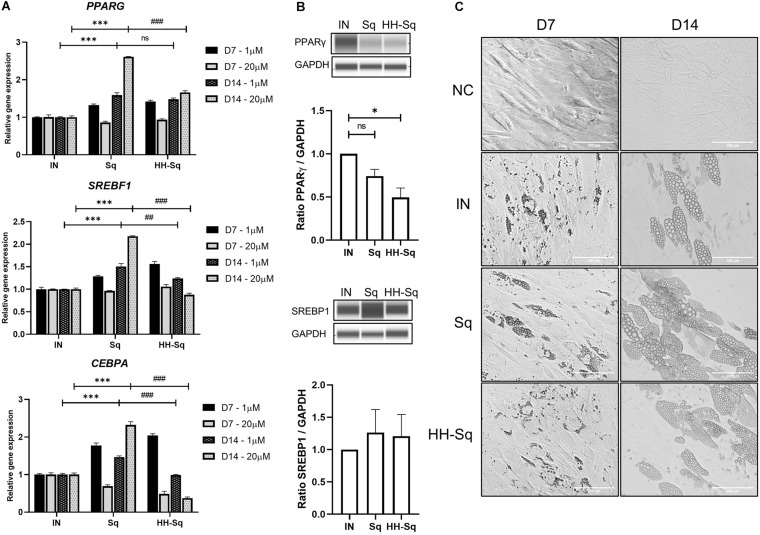
Relative expression level of adipocyte differentiation transcription markers and phenotypes. **(A)** Relative gene expression levels of *PPARG*, *SREBF1*, and *CEBPA*, normalized to *GAPDH* as endogenous control, treated with 1 and 20 μM at D7 and D14. Data are shown as mean ± SEM (*n* = 3). ns, non-significant. ***p < 0.001 vs. IN, ^##^*p* < 0.01 and ^###^*p* < 0.001 vs. Sq statistical difference was calculated using one-way ANOVA followed by Tukey test. **(B)** Representative protein bands of PPARγ and SREBP1 at D14 in adipocytes treated with 1 μM Sq and HH-Sq. Corresponding peak areas were normalized to the level of GAPDH, and relative ratios compared to IN were shown. ns, non-significant and *p < 0.05 vs. IN. **(C)** Representative light microscopic images at the D7 and D14 after the adipocyte induction. Scale bar indicates 100 μm. NC, no induction; negative control; IN, adipocyte-induced positive control; Sq/HH-Sq, adipocyte-induced treated with Sq and HH-Sq.

Obesity- and diabetes-induced cellular dysfunctions affect adipose tissue-derived stem cell proliferation and differentiation capacity causing reduced turnover in adipocytes, ectopic fat accumulation, and reduced number of preadipocytes (Bost et al., 2005; Van Tienen et al., 2011; Oñate et al., 2012). Thus, we measured gene expression of transcription factors that control adipogenesis in order to, first, validate the preadipocyte commitment of dASCs, and second, the effect of Sq and HH-Sq on those proadipogenic markers, which direct the differentiation. SREBP1c, peroxisome proliferator-activated receptor γ (PPARγ), and C/EBPα are early adipogenic markers acting synergistically on the downstream gene expression (Cawthorn et al., 2012). Knockdown of *SREBF1* inhibited adipogenesis and blocked the expression of the other two early markers (Ayala-Sumuano et al., 2011). Consequently, it was found that Sq treatments augmented gene expression of *PPARG*, *SREBF1*, and *CEBPA* compared to non-treated adipocyte-induced control (IN) (Figure 4A). However, the level of these gene expressions in HH-Sq-treated cells except *PPARG* remained inferior to that of Sq treatment, and it decreased in a time- and dose-dependent manner. Protein level of these transcription factors also remained lower in HH-Sq-treated adipocytes (Figure 4B). Of note, the effect of HH-Sq at D7 was comparable to that of Sq at D14 for *PPARG* and *SREBF1* and even higher in *CEBPA*, suggesting there is a temporal shift in the exertion of biological effect between these compounds. General aspect of adipocytes in differentiation at D7 and D14 is shown in Figure 4C. Smaller LDs were found in HH-Sq, whereas Sq-treated cells showed larger, comparable to IN, droplets that were most likely due to up-regulation of early adipogenic factors.

### HH-Sq Induced Adipocytes With Fewer Lipid Droplets

**FIGURE 5:**
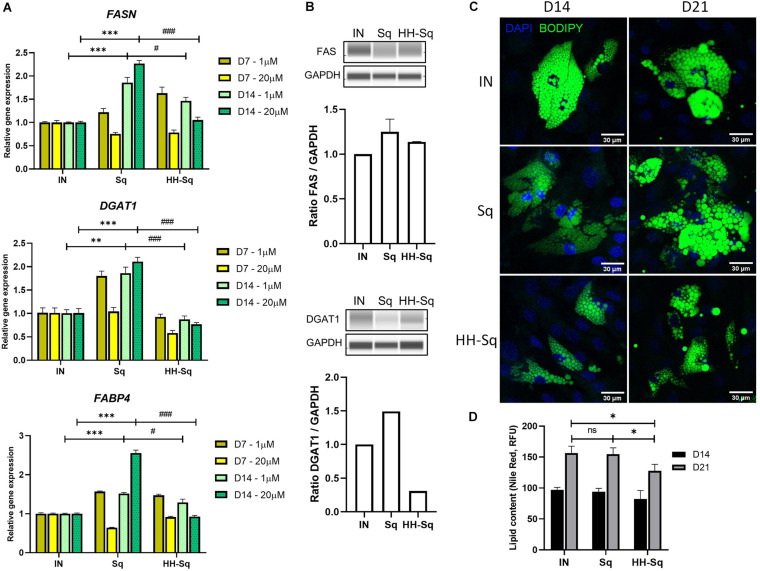
Expression of adipogenic markers and lipid droplet (LD) accumulation after adipocyte induction in dASCs. **(A)** Relative gene expression levels of *FASN*, *DGAT1*, and *FABP4*, normalized to *GAPDH* as endogenous control, treated with 1 and 20 μM at D7 and D14. **p < 0.01, ***p < 0.001 vs. IN, and ^#^*p* < 0.05 and ^###^*p* < 0.001 vs. Sq statistical difference was calculated using one-way ANOVA followed by Tukey test. **(B)** Representative protein bands of FAS and DGAT1. Corresponding peak areas were normalized to GAPDH, and relative ratios compared to IN were shown. **(C)** Representative fluorescence images of adipogenesis-induced dASCs treated with 1 μM of Sq and HH-Sq. At D14 and D21 after adipogenesis induction, LDs were stained with BODIPY 493/503 (green) and nucleus counterstained with DAPI (blue). Scale bar indicates 30 μm. **(D)** Lipid content was quantified by relative fluorescence unit (RFU) of Nile red staining (*n* = 4–5). IN-adipocyte-induced, non-treated control. Data are shown as mean ± SEM (*n* = 3). ns, non-significant. *p < 0.05 statistical difference was calculated using one-way ANOVA followed by Tukey test.

Committed preadipocytes undergo maturation by accumulating LDs as one of their primary roles and expressing subsequent late marker genes of lipogenesis and lipolysis. First, we checked downstream adipocyte-specific gene expression after D7 and D14 (Figure 5A). The fatty acid-binding protein 4 (*FABP4*), also known as adipocyte protein 2), is believed to be expressed in preadipocytes and mature adipocytes and thus required for the maturation of differentiation (Shan et al., 2013). Treatment with Sq and HH-Sq overexpressed *FABP4* compared to IN, showing a similar pattern as early transcription markers; however, increased level in HH-Sq was significantly lower than that in Sq. Next, we measured gene expression of enzymes required for the formation of LDs. Fatty acid synthase (*FASN*) and diacylglycerol acyltransferase (*DGAT1*) are key lipogenic enzymes necessary for triacylglycerol synthesis and LD formation (Schmid et al., 2005; Harris et al., 2011). Interestingly, both gene expressions were increased after D14 in Sq treatment, whereas only lesser extent for *FASN* not for *DGAT1* by HH-Sq treatment. Corresponding protein levels at D14 showed the similar tendency, however, the difference did not reach statistical significance (Figure 5B). Higher dose seemed to potentiate the effect of compounds, showing more increased expression of these genes by Sq and decreased by HH-Sq.

To extend this result, we observed the effect of treatments on the formation of LD; thus, cells were stained with a dye BODIPY at D14 and D21 after differentiation to visualize intracellular neutral lipids. A large number of newly formed LP appeared in the IN after D14, and its intensity and droplet size became more important by D21 (Figure 5C). Formed LD in Sq-treated cells seemed inferior after D14 of induction, but at D21, cells were engorged with lipids as much as observed in IN, and some cells showed slightly larger LD than IN. By contrast, LD density and size remained evidently fewer and smaller within the HH-Sq-treated cells. We also assessed the fluorescence intensity of lipids stained with Nile red dye. As observed in microscopic images, lipid content was significantly lower in HH-Sq-treated cells, whereas the lipid intensity was not different in Sq compared to IN up to D21 (Figure 5D). This suggests that the reduced presence of LD in HH-Sq-treated cells is in part due to inhibition of transcription factors but also due to partial inhibition of lipogenic enzymes.

### HH-Sq and Sq Increased Expression of Genes Involved in Metabolism

**FIGURE 6:**
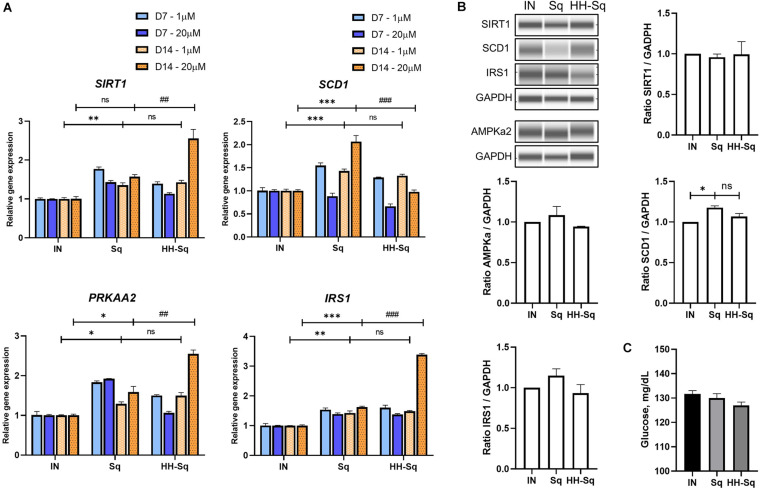
Metabolism related gene expression and protein levels in differentiated dASCs. **(A)** Relative gene expression of *SIRT1*, *SCD1*, *PRKAA2*, and *IRS1*, normalized to *GAPDH* as endogenous control, treated with 1 and 20 μM at D7 and D14. ns, non-significant. *p < 0.05, **p < 0.01, ***p < 0.001 vs. IN, and ^##^*p* < 0.01 and ^###^*p* < 0.001 vs. Sq statistical difference was calculated using one-way ANOVA followed by Tukey test. **(B)** Representative protein bands of SIRT1, SCD1, IRS1, and AMPKa2. Corresponding peak areas were normalized to GAPDH, and relative ratios compared to IN were shown. IN, adipogenesis-induced non-treated control. Data are shown as mean ± SEM (*n* = 3). ns, non-significant. *p < 0.05 statistical difference was calculated using one-way ANOVA followed by Tukey test. **(C)** Glucose level measured in supernatant of differentiated dASCs at D14 in the presence of 11 mM (198 mg/dL) glucose and 1 nM human recombinant insulin.

NAD-dependent deacetylase (*SIRT1*), a member of SIRTuins, has been suggested to be down-regulated in insulin-resistant tissue, and its activation improved insulin sensitivity (Sun et al., 2007). In addition, stearoyl-CoA desaturase 1 (*SCD1*), catalyzer in monosaturated fatty acid synthesis, is a key enzyme in *de novo* lipogenesis. Although gene expression of *SIRT1* and *SCD1* showed up-regulation in differentiated adipocytes exposed to 1 μM Sq and HH-Sq, higher concentration (20 μM) of HH-Sq stimulated the up-regulation of *SIRT1* and down-regulation of *SCD1* by D14, whereas Sq treatment showed the opposite effect (Figure 6A).

AMPK regulates a wide range of metabolic activities and activates *SIRT1* expression and its impairment involved in metabolic syndrome associated pathways (Ruderman et al., 2013). Thus, we measured the expression level of AMP-activated protein kinase (AMPK, gene *PRKAA2*) and insulin receptor substrate 1 (*IRS1*) as important markers of insulin sensitivity. Consistently, *PRKAA2* and *IRS1* genes were found to be overexpressed to the same extent in both treated cells at 1 μM, and this change was highly significant at 20 μM of HH-Sq (Figure 6A). Corresponding protein levels in cells treated at 1 μM were not significant (Figure 6B). After 14 days of adipogenic differentiation, we measured the glucose uptake rate in presence of insulin. The glucose concentration present in the supernatant of HH-Sq was slightly lower than Sq and IN (Figure 6C), suggesting improved glucose uptake. These observations indicate that HH-Sq prevented more effectively overactivation of lipogenesis, and in turn, more metabolic gene expressions were enhanced.

### Mitochondrial Biogenesis Improved by Sq and HH-Sq Treatment

**FIGURE 7:**
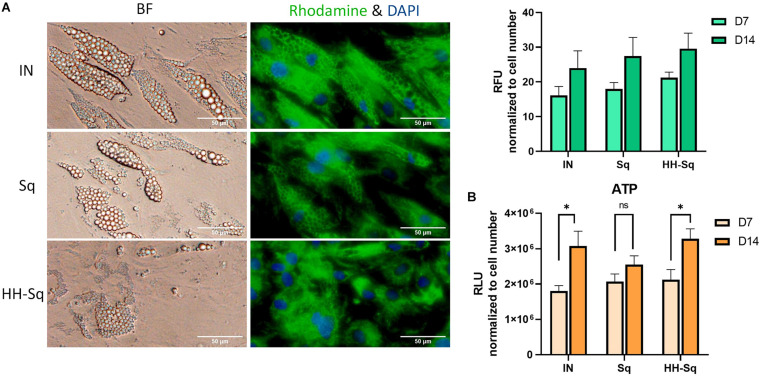
Mitochondrial biogenesis and ATP production in dASCs after D14 adipocyte differentiation. **(A)** Representative images captured in bright field (BF) and fluorescence microscope showing mitochondrial biogenesis at D14 stained with rhodamine 123 (green) and nucleus counterstained with DAPI (blue). Scale bar indicates 50 μm. Relative fluorescence unit (RFU) of rhodamine 123 was measured in 1 μM Sq and HH-Sq treatments. **(B)** ATP production was measured in dASCs after adipocyte differentiation. ns, non-significant. *p < 0.05 statistical difference was calculated using paired *t* test within group comparison at two different timepoints.

Mitochondria play an important role in energy homeostasis, apoptosis, and inflammation. In adipose tissue, mitochondrial metabolism and function are correlated to adipocyte differentiation, lipid metabolism, and insulin sensitivity (De Pauw et al., 2009). Therefore, mitochondrial dysfunction is widely reported to be implicated in metabolic diseases, obesity, and T2D (McBride et al., 2006; Green et al., 2011). Adipogenic transcription factors contribute in mitochondrial biogenesis during adipogenesis (Spiegelman et al., 2000). Thus, we evaluated mitochondrial biogenesis and ATP production in differentiated adipocytes at D7 and D14. As shown in Figure 7A, mitochondrial content increases throughout the adipogenesis from D7 to D14 in all groups. In Sq- and HH-Sq-treated cells, mitochondria remained nevertheless slightly augmented compared to IN. Mitochondrial proliferation in HH-Sq was accompanied by increased ATP production at D14 (Figure 7B), suggesting the process of adipogenesis and energy metabolism were concomitantly stimulated. This finding of mitochondrial content in Sq was not correlated to the level of ATP content, which increased to a lesser extent from D7 to D14.

**FIGURE 8:**
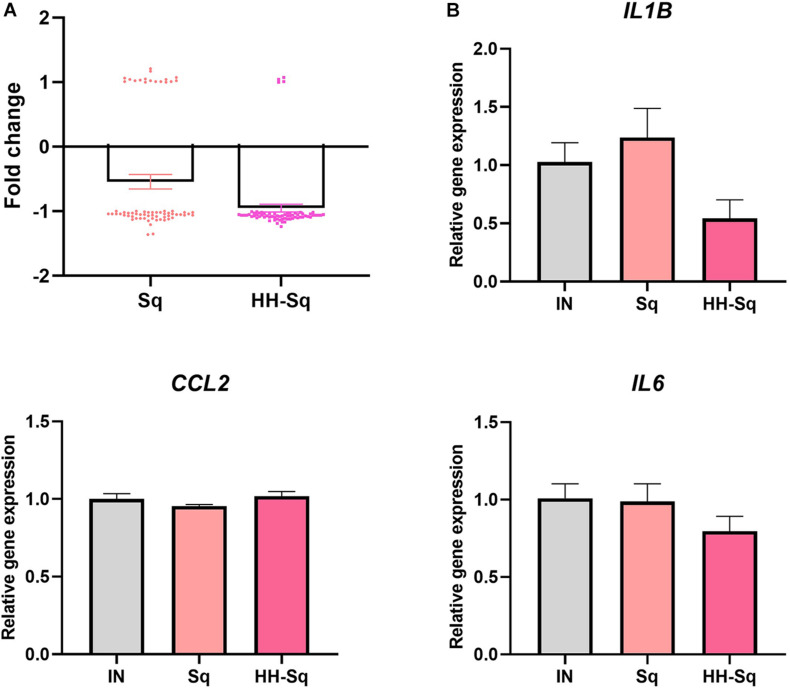
Inflammatory cytokine gene expression in dASCs after D14 adipocyte differentiation. **(A)** Cytokines (*n* = 66 genes) gene expression pattern with median averaging selected from microarray data. **(B)** Relative gene expression of *IL6*, *IL1B*, and *CCL2*, normalized to *GAPDH* as endogenous control, treated with 1 μM Sq and HH-Sq until D14.

Mitochondrial biogenesis alteration and its dysfunction are in part attributed to inflammatory or pathologic condition, which further leads to development of metabolic diseases and insulin resistance (Kim et al., 2008). Thus, we sought to analyze the changes in inflammatory cytokines in correlation with the treatment of Sq and HH-Sq during adipogenesis. Cytokine gene expression pattern of microarray data revealed a clear correlation of HH-Sq treatment and the number of down regulated inflammatory cytokines, although both Sq and HH-Sq prevented overexpression of a large number of cytokines (Figure 8A; list of genes in Supplementary Material). Gene expression levels by quantitative polymerase chain reaction showed slightly ameliorated tendency in HH-Sq-treated cells; however, none of *IL6*, *IL1B*, and *CCL2* reached a statistical significance (Figure 8B).

## Discussion

Pathogenic fat cells turn against their initial role in organism contributing to metabolic disorders such as obesity, T2D, and cardiovascular diseases through insulin resistance, an increase of free fatty acids, and impaired adipokines secretion (Van Gaal et al., 2006; Skurk et al., 2007). To compete with old hypertrophied pathogenic adipose tissue (Skurk et al., 2007) emitting undesired biological effects, researchers have been seeking more functional and responsive compounds that can promote new adipocyte differentiation, while not depleting the number of stem cells in reserve (McLaughlin et al., 2007; Ghaben and Scherer, 2019).

Adipose-derived stem cells have the properties of self-renewal and migration (Mazini et al., 2020). ASCs derived from subjects with diabetes showed the similar migratory and proliferative capacity in the absence and presence of Sq and HH-Sq, suggesting that the dASCs maintained the stem cell features, and both compounds exhibited neither depletion of stem cells nor interference on proliferation and migration.

Observations from global microarray data such as up- and down-regulated genes following the treatment with Sq and HH-Sq showed, respectively, different patterns in the number of genes and the process compared to that of commonly regulated genes. Despite this, MAPK cascade and protein serine-threonine kinase activity were commonly up-regulated. MAPK cascade is an important effector activated by extracellular stimuli to induce a long-term cellular response by triggering transcription of genes in the nucleus.

After adipocyte differentiation at D14, various genes involved in transcriptional regulation of adipogenesis differentiation were induced by Sq and HH-Sq treatment. For example, early markers, *PPARG*, *SREBF1*, and *CEBPA*, were overexpressed by Sq and lesser extent by HH-Sq. This ratio was preserved in the downstream lipogenic gene expression. Sq overactivated *FABP4*, *DGAT1*, and *FASN*, whereas their increased level of expression by HH-Sq was significantly lower compared to Sq. Furthermore, the observed effect of HH-Sq was more important at a higher dose. The resulting changes could be observed in LD size and intensity. In HH-Sq-treated adipocytes, the lipogenic genes were down-regulated with the formation of less and smaller LD up to D21 after differentiation.

The role of SCD1 is still controversial. SCD1 deficiency reduced lipogenesis and improved insulin sensitivity in adipose tissue (Flowers and Ntambi, 2008; Ralston and Mutch, 2015); however, SCD1-deficient mice have altered fatty acid re-esterification and glyceroneogenesis in white adipose tissue (Dragos et al., 2017). In Sq- and HH-Sq-treated cells, *SCD1* gene expression was increased but did not show any aberrant lipid accumulation in adipocytes. Hui et al. (2017) reported that mice with adipose tissue specific ablation of *SIRT1* are more susceptible to insulin resistance and argued adipocyte SIRT1 plays an important role in glucose homeostasis through modulation of macrophages. We observed a significant increase of *SIRT1* in Sq- and HH-Sq-treated adipocytes, which suggests improved glucose homeostasis.

Moreover, studies reported that AMPK is an important sensor of metabolic changes, and its level is highly reduced in insulin-resistant and morbidly obese subjects (Xu et al., 2012; Desjardins and Steinberg, 2018) and is activated in response to energy metabolism through AMPK-SIRT1-PCG-1α pathway to enhance mitochondrial oxidative function in adipocytes (Chau et al., 2010). In addition, IRS1 is known as a major insulin receptor substrate in mediating insulin action, and Rondinone et al. (1997) found that the level of IRS1 was significantly reduced in adipocytes of subjects with non-insulin-dependent T2D. Despite increased mitochondrial content both in Sq and HH-Sq indicates the induction of adipogenesis, ATP production level in Sq remained lower by D14. It might be inferred that more ATP was consumed for lipogenesis compared to HH-Sq, which is consistent with the results of overexpressed lipogenic genes followed by Sq treatment. Increased mitochondrial biogenesis and ATP production in HH-Sq-treated cells and the amelioration of glucose uptake in correlation with increased expression of *IRS1* can provide all collectively the benefic effect of HH-Sq in regulation of energy metabolism in adipocytes and its differentiation.

Local inflammatory status in adipose tissue systematically attracts macrophages and leukocytes as well as transforms resident macrophages into an activated form, which collectively aggravates insulin resistance and system inflammation (Chawla et al., 2011; Russo and Lumeng, 2018). Down-regulation of genes related to cytokine production and cytokine-mediated pathway by HH-Sq treatment also suggests that HH-Sq may have an anti-inflammatory effect because low-grade inflammation has been reported being increased in adipocytes of obese subjects due to the proinflammatory cytokines (Lasselin et al., 2014), which affect the pathogenicity of adipocytes (Ghaben and Scherer, 2019). Selected cytokine gene expression data from microarray (*n* = 66 genes) including individual gene expression levels (*IL6*, *IL1B*, and *CCL2*) tended to be ameliorated by HH-Sq treatment, whereas Sq-treated cells were not different than non-treated cells. However, the direct effect on adipocytes was not statistically significant; several protective effects might be stimulated. HH-Sq may improve inflammatory status in systemic level as adipose tissue macrophages are also inflammatory and contribute to adipocyte inflammation. This is supported by Sasaki et al. (2020) findings showing the anti-inflammatory effect of HH-Sq in murine macrophage model.

HH-Sq was synthesized from Sq by adding monoethylene glycol moiety with the aim of improving its hydrophilicity; however, the compounds actually exhibited difference at gene expression level in adipocyte differentiation. In particular, transmembrane receptor protein tyrosine kinase pathway and enzyme-linked receptor pathway genes were enriched by Sq treatment, whereas intracellular signal transduction and transduction by phosphorylation activities were specifically activated by HH-Sq as found by microarray analysis. Furthermore, the effect of HH-Sq on early adipogenic markers seems to precede that of Sq, showing comparable effect at D7 to Sq at D14. This suggests that HH-Sq might have penetrated cell membrane more actively than Sq and exerted its effect faster, which may, in part, explain the increased intracellular signaling genes in HH-Sq-treated cells. Intracellular signaling is necessary for long-term effect at the nucleus level by initiating gene expression, rather than short-term effect in cytoplasmic level.

In contrast, processes related to glucose transport and insulin signaling such as cytoskeleton-dependent intracellular transport (GO:0030705), response to insulin (GO:0032868), regulation of glucose import (GO:0046324), and response to growth factor (GO:0070848) were down-regulated in Sq-treated cells, suggesting Sq does not only weakly reach intracellular environment but also affect insulin signaling effector molecules at probably cell membrane level.

According to the current findings, it could be assumed that both Sq and HH-Sq exert an adipogenic effect on dASCs, and HH-Sq specifically enhances energy metabolism and insulin signaling without aberrantly activating lipogenesis during adipocyte differentiation. Effect of HH-Sq on ASCs derived from altered microenvironment can contribute to the resolution of insulin resistance and obesity-associated metabolic disorders, and the further development of an alternative class of therapeutics derived from a natural compound, and application in tissue engineering of fat. Nevertheless, future research should explore the molecular mechanism by which HH-Sq triggers intracellular signaling.

## Data Availability Statement

The datasets presented in this study can be found in online repositories. The names of the repository/repositories and accession number(s) can be found below: https://www.ncbi.nlm.nih.gov/, GSE153391.

## Author Contributions

MG: conceptualization, methodology, investigation, visualization, validation, and original draft writing. MG and FF: formal analysis and data curation. FF: performed microarray experiment. TA and KT: synthesized HH-Sq. FF, HI, and TA: writing review & editing. KT and HI: supervision and funding acquisition. All authors reviewed the manuscript.

## Conflict of Interest

The authors declare that the research was conducted in the absence of any commercial or financial relationships that could be construed as a potential conflict of interest.

## Footnotes

1 https://david.ncifcrf.gov/

2 https://www.gsea-msigdb.org

3 www.ncbi.nlm.nih.gov/geo
